# *Chaetomium globosum*: Spotting a scarce saprophyte in bacterial-fungal co-infection of the sinuses in a post-COVID-19 patient

**DOI:** 10.1016/j.mmcr.2023.100618

**Published:** 2023-12-03

**Authors:** Neeta Gade, Soumyabrata Nag, Kiran Kumar Prathipati, Meena Mishra, Vishal Shete

**Affiliations:** a Department of Microbiology, AIIMS Nagpur, MIHAN, Nagpur, 441108, India; b Department of ENT, AIIMS Nagpur, MIHAN, Nagpur, 441108, India

**Keywords:** *Chaetomium globosum*, Post COVID-19, Perithecia, Ascocarp, Fungal co-infection

## Abstract

Atypical fungal co-infections in post-COVID-19 patients may have been underreported due to limited diagnostic methods. We present a case of *Chaetomium globosum* sinusitis in a 55-year-old post-COVID-19 patient with pain in the left side of the face, mimicking rhino-cerebral mucormycosis.

CT-paranasal sinuses showed mucosal thickening of left paranasal sinuses, biopsy of which grew a velvety, white colony. It was confirmed as *Chaetomium globosum*. The patient responded to oral Posaconazole therapy for three months.

Prompt identification of atypical fungal agents is critical for appropriate treatment.

## 1 Introduction

The world was fighting against the life-threatening complications of coronavirus disease (COVID-19) and its ever-emerging variants. Due to the widespread use of broad-spectrum antibiotics, corticosteroids, and invasive or non-invasive ventilation in the treatment of the pandemic, this novel coronavirus paved the way for opportunistic fungal infections in post-COVID-19 patients [1]. While mucormycosis gained particular attention during the second wave of COVID-19, other secondary fungal co-infections may have been neglected or underreported due to limited diagnostic methods. We present a clinical case of bacterial-fungal co-infection of the sinuses in a post-COVID-19 patient with *Chaetomium globosum* and multidrug-resistant *Escherichia coli.*

## 2 Case

A 55-year-old patient, presented to the ENT OPD at AIIMS, Nagpur, complaining of continuous pain involving the left periorbital region, left jaw, and left ear for 15 days duration. The patient was admitted (Day 0) with clinical suspicion of mucormycosis, as there was a recent history of mild COVID-19 one month prior (Day −32), requiring only home isolation (for 14 days), but with an on-and-off headache from then onwards. On ENT examination there was mucinous secretion coming from the left maxillary sinus. The rest of his PNS (para-nasal sinus) examination was within normal limits. A computerized tomography scan (CT scan) of PNS, done on the next day (Day +1), showed mucosal thickening involving the left maxillary, bilateral ethmoid, frontal and sphenoid sinuses. The patient underwent Functional Endoscopic Sinus Surgery (FESS) on Day +3.

Pus samples and biopsy tissue collected during FESS were immediately sent to the microbiology laboratory. It was processed for bacteriological and fungal microscopy and culture. The samples were inoculated on Blood and MacConkey's agar for bacterial culture and plates were incubated at 37 °C. For fungal microscopy, part of the sample was inoculated in 10 % KOH, and for culture, the sample was inoculated on two sets of Sabouraud's dextrose agar with chloramphenicol (SDA) and incubated at 25 °C and 37 °C. The 10 % KOH mount of the sample showed fragments of hyaline fungal hyphae. Bacterial culture yielded the growth of multidrug-resistant *Escherichia coli*, for which the antibiotic was changed from Ceftriaxone (given 1 gm IV, twice daily from Day 0 to +5) to Doxycycline (100 mg oral tablets, twice daily) and Piperacillin-Tazobactam (4.5 gm IV, thrice daily), administered for the next 10 days (Day +6 to Day +15).

**Fig. 1:**
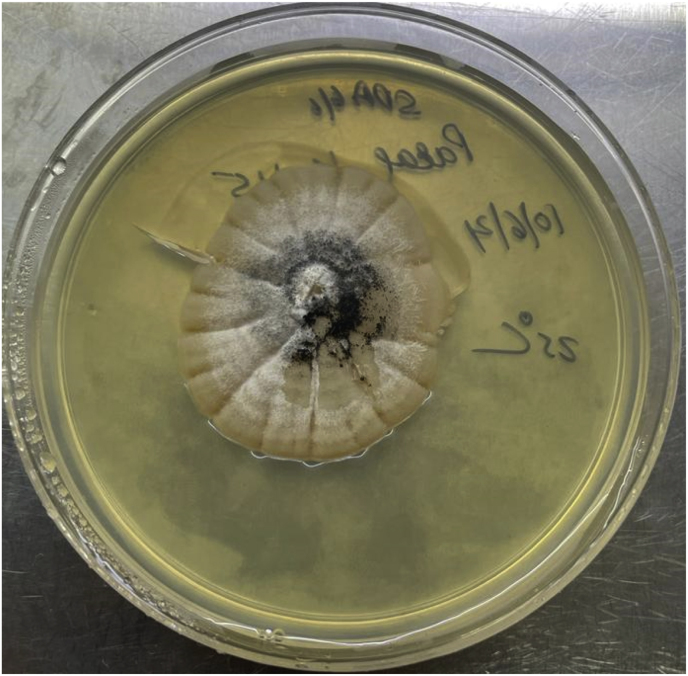
Sabouraud's dextrose agar plate showing irregular, corrugated, white cottony colony, with radial striations, of *Chaetomium globosum*.

**Fig. 2:**
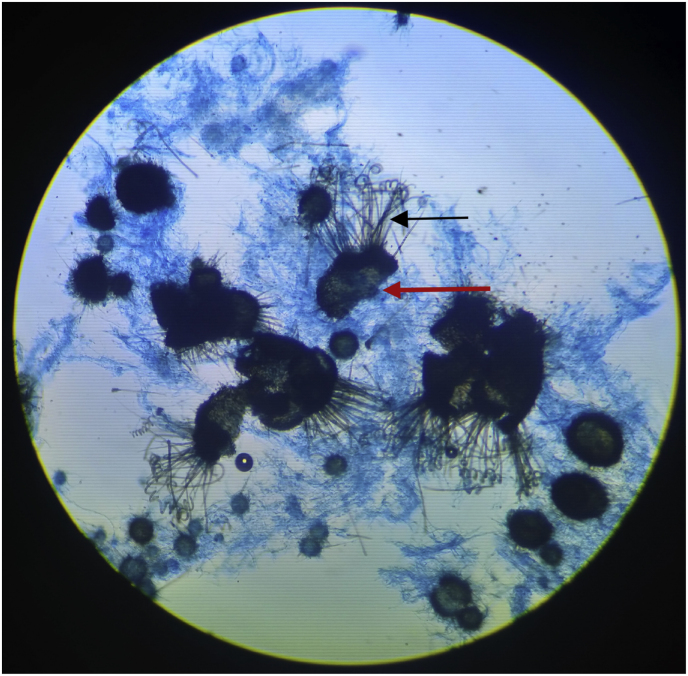
LPCB mount (10x) showing hyaline septate hyphae and dark brown spherical ascoma or perithecia (red arrow) with numerous hairs (black arrow).

A small velvety, white colony with reverse buff was grown on SDA after seven days of incubation at 25 °C, which was further incubated for another week for maturation of fungal structures [Fig. 1]. The growth's lactophenol cotton blue (LPCB) mount showed septate hyphae and perithecia asci. Ascocarps showed numerous brown to black perithecia covered with long hair-like dematiaceous setae [Fig. 2] and the isolate was identified as *Chaetomium* species (Day +17), which was reconfirmed as *Chaetomium globosum* (Day +62) from the National Mycology Reference Laboratory, Postgraduate Institute of Medical Education and Research, Chandigarh. Initially, IV Amphotericin B (Intralipid/lipid emulsion), 350 mg IV, once daily, was started from Day +6 based on clinical suspicion of mucormycosis, but after 3 doses, on Day +8, the patient developed allergic skin lesions and thus was shifted to oral Posaconazole tablets from Day +9. The first dose was 600 mg followed by 300 mg, once daily from the next day. The patient improved clinically and was discharged after two weeks with the advice to continue oral Posaconazole for three months. The patient improved clinically on follow-up examination (D +95).

## 3 Discussion

*Chaetomium* species belong to a large genus of saprophytic ascomycetes found in soil and on dung, straw, seeds, and plant debris. Although *Chaetomium* species are rarely implicated in human disease, their spectrum of mycoses includes onychomycosis and sinusitis in immunocompetent individuals and empyema, pneumonia, and fatal disseminated cerebral disease in immunocompromised hosts and intravenous drug users [[2], [3], [4], [5]]. Rarely it has been reported to cause endocarditis after repair of the native valve [6]. *C. globosum* has been implicated in most of the clinical cases followed by *Chaetomium atrobrunneum*. Another species, *Chaetomium strumarium* along with *C. atrobrunneum* is now believed to cause primary brain abscess which previously was attributed only to *C. globosum* [4,7]. COVID-19 patients experience immuno-suppression with a decrease in CD4^+^ T and CD8^+^ T cells and overexpression of inflammatory cytokines increasing their vulnerability to opportunistic infections by creating an environment conducive for fungal and bacterial coinfections [8]. Research suggests that viral respiratory diseases like COVID-19 may predispose an individual to other fungal and bacterial coinfections and superinfections [9,10]. The clinical presentation in our patient was initially suspected as mucormycosis, but culture growth revealed the presence of *Chaetomium* species which was later confirmed as *Chaetomium globosum*, and multidrug-resistant *E. coli*.

The relative lack of sporulation structures coupled with the gradual formation of the Ascomata fruiting bodies in *Chaetomium,* makes morphological identification difficult, posing routine identification a diagnostic challenge. Often in cases of brain abscesses the etiologic agent was identified as a *Chaetomium* species after the patient's death [2]. Knowledge about the susceptibility of *Chaetomium* to conventional antifungal agents is also sparse. A study by Guarro et al. [11]*,* in 1995, on 24 strains of *Chaetomium* species including *C. globosum*, indicated that they are resistant to flucytosine and fluconazole, while inhibitory activity was exhibited by azoles like itraconazole and ketoconazole, but no anti-fungal agent demonstrated fungicidal activity. The appropriate treatment for *Chaetomium* infections is thus unknown. Further, there are no recommended antifungal testing methods or management strategies with antifungal agents for *Chaetomium* species. In our case, the patient developed a reaction to Amphotericin B which led to a change of antifungal to Posaconazole to which the patient responded.

The simultaneous presence of *E. coli* and a fungal pathogen in the sinuses in a post-COVID-19 patient presents a complex and troubling clinical situation. Distinguishing symptoms arising from co-infections and detecting them is a complex task, and clinicians may need to utilize advanced diagnostic methods like PCR tests, culture studies, or medical imaging to accurately identify them. Microbiologists also should not disregard the possibility of co-infection simply because a bacterial pathogen has grown in culture.

The above case highlights the importance of obtaining microbiological cultures as part of the routine management of patients suspected of having a fungal infection. It also generates awareness among clinicians and diagnostic laboratories regarding the identification and management of *Chaetomium*-associated diseases.

## Conflict of interest

There are none.
